# Young children with Noonan syndrome: evaluation of feeding problems

**DOI:** 10.1007/s00431-020-03664-x

**Published:** 2020-05-11

**Authors:** Jos M. T. Draaisma, Joris Drossaers, Lenie van den Engel-Hoek, Erika Leenders, Joyce Geelen

**Affiliations:** 1 Radboud Institute for Health Sciences, Amalia Children’s Hospital, Department of Pediatrics, Radboud University Medical Center, Nijmegen, The Netherlands; 2 Donders Institute for Brain, Cognition and Behaviour, Department of Rehabilitation, Radboud University Medical Center, Nijmegen, The Netherlands; 3 Department of Human Genetics, Radboud University Medical Center, Nijmegen, The Netherlands

**Keywords:** Noonan syndrome, Early onset feeding problems, Late onset feeding problems, Gastroesophageal reflux disease, Dysphagia

## Abstract

Noonan syndrome (NS) is a common genetic syndrome with a high variety in phenotype. Even though genetic testing is possible, NS is still a clinical diagnosis. Feeding problems are often present in infancy. We investigated the feeding status of 108 patients with clinically and genetically confirmed NS. Only patients with a documented feeding status before the age of 6 were included. A distinction was made between patients with early onset feeding problems (< 1 year) and children with late onset feeding problems (> 1 year). Seventy-one of 108 patients had feeding problems, of which 40 patients required tube feeding. Children with a genetic mutation other than *PTPN11* and *SOS1* had significantly more feeding problems in the first year. Fifty-two of all 108 patients experienced early onset feeding problems, of which 33 required tube feeding. A strong decrease in prevalence of feeding problems was found after the first year of life. Fifteen children developed feeding problems later in life, of which 7 required tube feeding.

*Conclusion*: Feeding problems occur frequently in children with NS, especially in children with NS based on genetic mutations other than *PTPN11* and *SOS1*. Feeding problems develop most often in infancy and decrease with age.

**Table:** 

**What is Known:** • *Young children with Noonan syndrome may have transient feeding problems.* • *Most of them will need tube feeding.*	
**What is New:** • *This is the first study of feeding problems in patients with clinically and genetically proven Noonan syndrome.* • *Feeding problems most often develop in infancy and resolve between the age of 1 and 2.*

**What is Known:**

• *Young children with Noonan syndrome may have transient feeding problems.*

• *Most of them will need tube feeding.*

**What is New:**

• *This is the first study of feeding problems in patients with clinically and genetically proven Noonan syndrome.*

• *Feeding problems most often develop in infancy and resolve between the age of 1 and 2.*

## Introduction

Noonan syndrome (NS) is a well-known congenital disorder with an estimated incidence of 1:1.000–2.500 live births [1]. NS is characterized by a short stature, typical facial dysmorphism and a wide spectrum of congenital heart defects [2–5]. NS is caused by germline mutations in the Ras-mitogen-activated protein kinase (RAS/MAPK) pathway called RASopathies [6]. At present-day, mutations in more than 16 genes functioning in the RAS/MAPK pathway are defined as a cause of NS. The most frequent mutations are activating germline gene mutations in the *PTPN11* (± 50%) and *SOS1* gene (±13%) [7]. In 18%–24% of the patients with a clinical diagnosis of NS no mutation is found [8, 9]. Clinical scoring systems, such as the van der Burgt Criteria, have been developed to aid the diagnostic process [1]. Major and minor criteria, such as facial dysmorphism, heart defect, body length and other criteria, are part of the van der Burgt Criteria. Although feeding problems are not included or mentioned in the diagnostic criteria, they are described in up to 76% of patients with Noonan syndrome and appear to correlate with a delay in developmental milestones and poorer long-term outcome [10, 11]. The severity and course of feeding problems, the underlying cause and the association with the genotype has not yet been studied in more detail. The aim of this study was to give a more extensive overview of the severity and course of early onset (< 1 year of age) versus late onset (1–6 years of age) feeding problems in children with NS and its association to specific gene mutations. In addition, we aimed to identify the documented presumed or proven causes of these feedings problems in both groups to help health care providers to recognize and treat these problems in young patients with NS.

## Materials and methods

### Patient population

A retrospective cohort study of all patients (at that time) clinically diagnosed with NS or Noonan-like syndrome (both included in the term NS) from January 1995 to January 2019 was performed at the Radboudumc Amalia Children’s hospital. Patients without available data on the feeding history in the medical records or patients without genetic confirmation of the clinical diagnosis were excluded. The medical records were reviewed from birth through February 2019. The prevalence of feeding problems was calculated in four different age groups (0–1 year / 1–2 years / 2–4 years / 4–6 years). As we included patients until January 2019, not all patients reached the age of 6, so the groups of patients in the 1–2 year, 2–4 year and 4–6 year group will be smaller than the 0–1 year group.

To quantify the severity of the feeding problems, the validated Functional Oral Intake Scale was used [12, 13]. The 7 levels of the Functional Oral intake Scale were compromised into three categories (oral diet without restrictions (level 7), oral diet with restrictions (level 4–6) and tube-dependent feeding (levels 1–3)) to provide a comprehensive overview of the course of the feeding problems [13]. The feeding status of the children was linked to their genotype. Whenever possible, the documented presumed or proven cause of the feeding problems was extracted from the available data. We documented gastroesophageal reflux disease when complications were noted from gastroesophageal reflux, including feeding difficulties, hematemesis and poor weight gain, for which a therapeutic intervention was started.

### Statistical analysis

Data were analysed using SPSS statistic 24.0. Descriptive statistics were used for patients’ characteristics, prevalence and severity of feeding problems and the relation to the gene mutation. Categorical variables were described as number and percentage. Feeding problems were analysed according to the age of development: the first group consists of NS patients in whom the feeding problems developed in the first year of life. The second group consists of patients with NS in whom the feeding problems developed between age 1 and 6 years. For statistical analysis, the NS patients were divided in three groups with different gene mutations: *PTPN11*, *SOS1* and the gene mutation group with low prevalence. This gene mutation group was created due to the expected small numbers of patients with other gene mutations. In order to compare the prevalence of feeding problems between these groups, Fisher’s exact test was used with a significance level of 0.05.

## Results

### Patient characteristics of the entire group

A total of 490 patients with Noonan **s**yndrome were diagnosed and/or treated at the Radboud University Medical **C**enter**,** of which 252 patients had genetically confirmed NS. There was no data available on feeding problems for 144 patients, leaving 108 patients (43 male**s** and 65 female**s**) with available data to be included in the four different age groups: 108 (0–1 year), 97 (1–2 years), 95 (2–4 years) and 79 (4–6 years). Of these 108 children, 37 (34%) never had feeding problems, 31 (29%) had an oral diet with restrictions and 40 (37%) were feeding tube**–**dependent at some time during the first 6 years of life. Of the 63 children with a *PTPN11* gene mutation, 40 (63%) children had feeding problems at any time during the first 6 years of life, of which 17 (43%) received an oral diet with restrictions and 23 (57%) children required tube feeding. Of the 16 children with a *SOS1* gene mutation, eight (50%) had feeding problems, of which six (75%) had an oral diet with restrictions and two (25%) children required tube feeding. Of the 29 children diagnosed with gene mutations with a low prevalence (*KRAS* (*n* = 6), *RAF1* (*n* = 4), *BRAF* (*n* = 4), *SHOC2* (*n* = 4), *HRAS* (*n* = 2), *CBL* (*n* = 3), *RIT1* (*n* = 3), *SOS2* (*n* = 2) and *MAP2K2* (*n* = 1)), 23 (79%) patients developed feeding problems, of which eight (35%) had an oral diet with restrictions and 15 (65%) received tube feeding (Table 1). There was no difference in the prevalence of feeding problems between the different genes involved (*PTPN11* vs *SOS1*: *p* = 0.39; *PTPN11* vs other gene mutations: *p =* 0.15, *SOS1* vs other gene mutations: *p =* 0.05).

**Table 1:** Overview of feeding problems in young children with Noonan syndrome with different gene mutations

	*PTPN11* (*n* = 63)	*SOS1* (*n* = 16)	Other gene mutations (*n* = 29)
Oral diet without restrictions	23 (36%)	8 (50%)	6 (21%)
Oral diet with restrictions	17 (27%)	6 (38%)	8 (27%)
Tube-dependent feeding	23 (37%)	2 (12%)	15 (52%)

### Early onset feeding problems

Of the 108 included children**,** 56 (52%) experienced early onset feeding problems. Of these 56 children, 23 (41%) had an oral diet with restrictions and 33 (59%) were tube**-**dependent. Sixteen (of which 12 tube**-**dependent) of these 56 children continued to have feeding problems after their first year of life. After 2 years 7 children remained (6 tube**-**dependent), and**,** after the age of 4, 3 children (2 tube-dependent) were still having feeding problems (Table 2). Of the 63 children diagnosed with a *PTPN11* mutation, 34 (54%) children had early onset feeding problems. Eight of these 34 children (of whom 6 tube-dependent) continued to have feeding problems after 1 year of life. Of those 8 children, 3 children were still having feeding problems after 2 years. After the age of 4, only 1 child remained having feeding problems.

**Table 2:** The course of early feeding problems in gene-mutation positive Noonan children

	Category of feeding problem	*PTPN11* (*n* = 63)	*SOS1* (*n* = 16)	Other genes (*n* = 29)
0–1 year	Oral diet without restrictions Oral diet with restrictions Tube-dependent feeding	27 16 20	10 4 2	15 3 11
1–2 years	Oral diet without restrictions Oral diet with restrictions Tube-dependent feeding	48 2 6	14 0 0	17 2 6
2–4 years	Oral diet without restrictions Oral diet with restrictions Tube-dependent feeding	51 1 2	13 0 0	23 0 4
4–6 years	Oral diet without restrictions Oral diet with restrictions Tube-dependent feeding	47 1 0	10 0 0	19 0 2

In this cohort, 6 of 16 (38%) children with a *SOS1* mutation (38%) had feeding problems in infancy. After the first year of life, none of these children were having feeding problems. There was no difference between the prevalence of feeding problems in any age group between the children with *PTPN11* and *SOS1* mutation (*p* = 0.27). However, the prevalence of feeding problems in infancy was 80% in the group patients with a gene mutation with low prevalence (*PTPN11* vs other gene mutations: *p =* 0.02, *SOS1* vs other gene mutations: *p <* 0.01) (see Table 3 for feeding problems in patients with other gene mutations). The feeding problems in children with *SOS1* mutation decreased significantly faster than children with other gene mutations (*p* = 0.03).

**Table 3:** The category of feeding problems in the first year of life in patients with Noonan syndrome with different gene mutations

	Category	PTPN11	SOS1	KRAS	RAF1	BRAF	SHOC2	HRAS	CBL	RIT1	SOS2	MAP2K2
*N=*		63	16	6	4	4	4	2	3	3	2	1
	No restrict With restrict Tube	27 16 20	10 4 2	5 0 1	2 2 0	2 0 2	3 0 1	0 0 2	1 0 2	1 0 2	1 0 1	0 1 0

Category, category of feeding problem; No restrict, oral diet without restrictions; With restrict, oral diet with restrictions; Tube, tube-dependent feeding

The presumed or proven cause of the feeding problems was explicitly documented in 40 of the 56 children with early onset feeding problems. Twenty-seven of the 40 children (68%) suffered from gastroesophageal reflux disease, and three children suffered from severe constipation together with feeding problems. There was no difference in the prevalence of the documented presumed or proven cause between the different groups (prevalence of gastroesophageal reflux disease: *PTPN11* vs *SOS1*: *p* = 1.00; *PTPN11* vs other gene mutations: *p =* 0.08, *SOS1* vs other gene mutations: *p* = 1.00).

### Late onset feeding problems

After the age of 1, 15 of 97 (15%) children developed feeding problems for the first time. Ten of these children developed feeding problems in the age group 1–2 years, one child in the age group 2–4 years and four children in the age group 4–6 years. Five children (9%) with *PTPN11* gene mutation developed late onset feeding problems, 2 after an intercurrent infection, 2 children post-operatively and 1 due to behaviour problems. Three children (21%) with a SOS1 gene mutation developed late onset feeding problems, one due to delayed gastric emptying, 1 because of an eating aversion caused by autism and 1 child due to a bad condition caused by heart disease. Seven children out of the group of 25 children (28%) with 1 of the other gene mutations developed late onset feeding problems: 1 child with a *SOS1* mutation due to gastroesophageal reflux disease, 1 child with a *SHOC2* mutation developed feeding problems mainly due to a 3MCC deficiency, 1 child with a *SHOC2* mutation due to malabsorption, 1 child with *SOS2* mutation due to the Dubin–Johnson syndrome, 1 child with *CBL* mutation due to juvenile myelomonocytic leukaemia, 1 child with *CBL* mutation due to cow’s milk allergy and 1 child with *CBL* mutation due to delayed gastric emptying. From these 15 children, 8 children were tube-dependent for some time.

## Discussion

In this retrospective study**,** the prevalence of feeding problems in children with genetically confirmed NS was assessed. Molecular testing identifies mutations in *PTPN11* in about 50%–60%, *SOS1* in about 15%, *RAF1* in 3–17% and the other genes in 5% or less of genetically proven patients with NS and NL syndrome [14]. In our population, we found the same distribution of type of mutation. The prevalence of feeding problems in the first year was 52% and more than half of our patients were tube-dependent. This is consistent with previous studies on this subject. Feeding problems in association with Noonan syndrome were already reported in 1992. In this study, more than 76% of patients with clinically diagnosed NS experienced feeding problems in infancy, of which almost one-third was tube-dependent [10]. In 1999, feeding problems were described in 64% of young children with clinically diagnosed NS [15]. All these children required tube feeding. Shaw et al. published in 2007 that 65% of children with clinically diagnosed NS experienced feeding problems in the first months of life, of which 36% were tube-dependent [11]. The most recent study on this subject, from Croonen et al., described feeding problems in 56% of children younger than 1 year old with clinically and genetically proven Noonan syndrome [16]. More than half of these children (59%) were tube-dependent. Our results are consistent with this recent paper. There was no difference in the prevalence of feeding problems in infancy in patients with a *PTPN11* or *SOS1* mutation. However, the prevalence of early onset feeding problems in patients with a gene mutation with low prevalence was much higher with the same documented presumed or proven cause.

The possible course of feeding problems was only mentioned in the study of Shah et al. [15]. They mentioned that the feeding problems remarkable improved after the age of 3 to 4. In our study**,** we found a major improvement of the feeding problems between the age of 1 to 2, with only a minority having still feeding problems after the age of 2. In patients with a *SOS1* mutation the feeding problems improved even significantly faster. When feeding problems persisted, children most oftenly stayed tube-dependent. The documented presumed or proven cause of the feeding problems was most often gastro-oesophageal reflux disease (68%). Gastro-oesophageal reflux disease was documented in 44% of children with feeding problems by Shah et al., as well as delayed gastric emptying [15]. At the present time, the diagnosis of gastroesophageal reflux disease can be made based on clinical symptoms. An upper gastro-intestinal study can be used for evaluating other anatomic causes of vomiting. Upper endoscopy is only considered for children with concerning symptoms, persistent symptoms despite treatment, and relapse of symptoms after treatment [17]. Delayed gastric emptying may exacerbate any tendency to gastroesophageal reflux disease [15]. Gastric emptying studies may play a role in the diagnosis of delayed gastric emptying [18]. We think, and this was also postulated by Shah et al. [15], that the cause of the feeding problems is a delayed gastrointestinal motor development and that this improves with age.

We could not study growth as a consequence of the feeding problems, due to inappropriate data. However, in an earlier study with another design, we showed that children with Noonan syndrome and feeding problems were clearly lighter and shorter and had a clinically relevant lower weight and length SDS at the age of 1 than children without feeding problems [16].

Remarkable was the fact that almost **1** out of **7** children developed feeding problems at an older age. This was frequently due to postoperative circumstances, infections or co-morbidities, and not directly due to the causes of early-onset feeding problems. However, it is known that infections, or (operations for) heart diseases and cancer are more common in children with NS [19–21]. So having Noonan syndrome could be an indirect factor of late onset feeding problems and children with NS might be prone to feeding problems that can be triggered by, for example, an infection. This is illustrated by the case report that delayed gastric emptying may even present for the first time during adulthood [22]. No children were diagnosed with celiac disease, although Quaio et al. notify that physicians should be alerted to the possibility of autoimmune diseases, including celiac disease, in patients with NS [23].

This study has some limitations. Due to the retrospective design, many patients had to be excluded. However, the included patients had the same distribution of type of mutation as in the literature, and the recorded prevalence was in the same range as in the literature. So, we think, the recorded prevalence data may be reliable. Another problem due to the retrospective design, is that follow-up data were limited and not always straightforward. Moreover, symptoms of the existing feeding problems were not always clearly documented, and the cause of the feeding problems was often not studied extensively. The scoring system used in the literature could not be used due to the retrospective design [10, 16]. For this reason, we used the validated Functional Oral Intake Scale [12, 13]. This scale may underestimate the prevalence of (behavioural) feeding problems, thus underestimating the prevalence. Moreover, there was no information on psychiatric comorbidity and/or behavioural problems in our retrospective data in these young children.

There is no literature on psychiatric/behavioural problems in young children (< 4–6 years) with Noonan syndrome. However, it is known that feeding problems in infants and young children are caused by different issues like medical and/or developmental history, problems with motor control and function, problems with swallowing and behaviour, that all may play a role and may interact with each other [24]. Nevertheless, this study is the first to combine prevalence of feeding problems, the course and presumed or proven causes of feeding problems and the association with the genetic mutation. Another limitation is the fact that, using the ClinGen gene curation framework, it can be questioned if the used diagnosis is correct [25]. So is an *HRAS-*mutation definitively associated with Costello syndrome, and is a *BRAF-*mutation moderately associated with NS and definitively associated with cardiofaciocutaneous syndrome. However, as these children are collected in the group other gene mutations, the results still may reflect the prevalence of feeding problems in patients with NS.

## Conclusion

Most children with clinically and genetically proven NS or Noonan-like syndrome have feeding problems from as early as infancy. Children with a gene mutation with low prevalence even have a higher risk. When feeding problems are present, tube feeding is often required in order to optimize the feeding status. These feeding problems may mainly be due to gastro-oesophageal reflux disease, and/or to delayed gastric emptying, and improve remarkably between the age of 1 and 2. However, feeding problems may develop later in life, which are mostly due to comorbidities. Given the high prevalence of feeding problems at an early age in children with NS, it is necessary to evaluate feeding problems in an evidence-based way to treat the feeding problems accurately in an evidence-based way.

## Abbreviations

NS: Noonan syndrome
RAS/MAPK: Ras-mitogen-activated protein kinase
